# Supplementary material for: Electron-momentum dependence of electron-phonon coupling underlies dramatic phonon renormalization in YNi2B2C
Source: Nat Commun. 2022 Jan 11;13:228. doi: 10.1038/s41467-021-27843-y (PMC8752669; doi:10.1038/s41467-021-27843-y)
Supplement: Supplementary file 1 — Supplementary Information [file 41467_2021_27843_MOESM1_ESM.pdf]

SUPPLEMENTARY INFORMATION TO:

Electron-momentum dependence of electron-phonon coupling underlies dramatic phonon renormalization in YNi_2B_2C

Philipp Kurzhals[1], Geoffroy Kremer[2], Thomas Jaouen[2,3], Chris W. Nicholson[2], Rolf Heid[1], Peter Nagel[1], John-Paul Castellan[1,4], Alexander Ivanov[5], Matthias Muntwiler[6], Maxime Rumo[2], Bjoern Salzmann[2], Vladimir N. Strocov[6], Dmitry Reznik[7,8], Claude Monney[2], Frank Weber[1,✉]

[1] Institute for Quantum Materials and Technologies, Karlsruhe Institute of Technology, 76021 Karlsruhe, Germany
[2] Département de Physique and Fribourg Center for Nanomaterials, Université de Fribourg, 1700 Fribourg, Switzerland
[3] Univ Rennes, CNRS, IPR (Institut de Physique de Rennes) - UMR 6251, F-35000 Rennes, France
[4] Laboratoire Léon Brillouin (CEA-CNRS), CEA Saclay, F-91911 Gif-sur-Yvette, France
[5] Institut Laue-Langevin, 71 avenue des Martyrs CS 20156, 38042 Grenoble Cedex 9, France
[6] Paul-Scherrer Institut, Swiss Light Source, 5232 Villigen PSI, Switzerland
[7] Department of Physics, University of Colorado at Boulder, Boulder, Colorado, 80309, USA
[8] Center for Experiments on Quantum Materials, University of Colorado at Boulder, Boulder, Colorado 80309, USA

✉ frank.weber@kit.edu

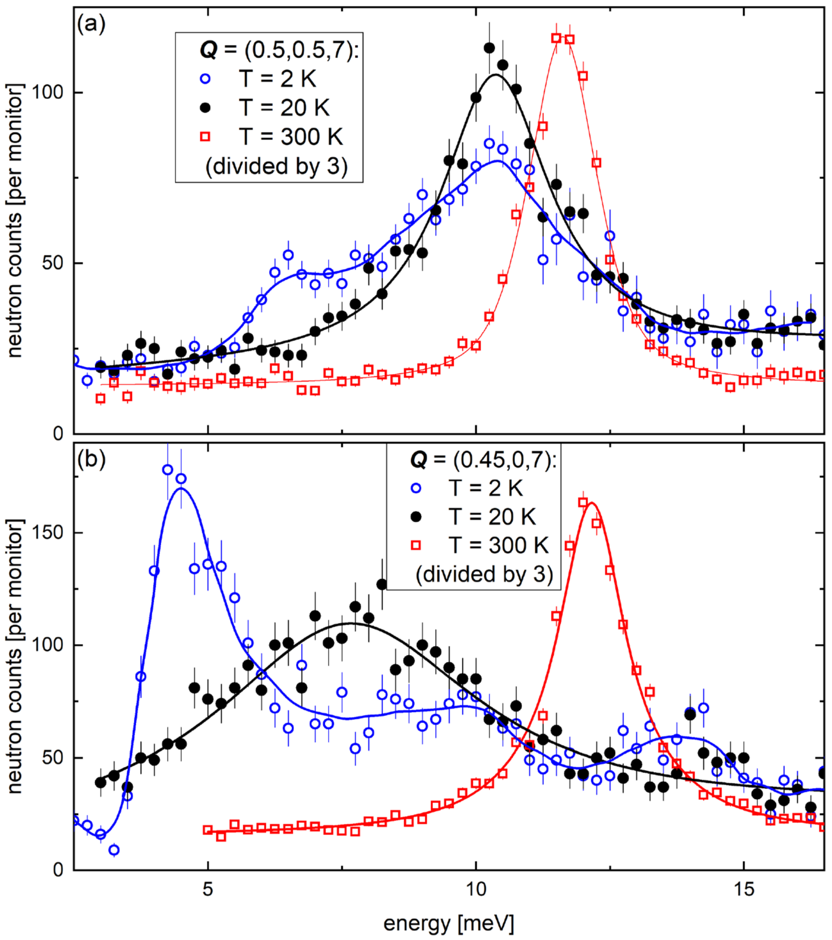

FIG. S1. T_c effect in phonon spectroscopy.

(a,b) Raw data from inelastic neutron scattering (INS) at (a) $\mathbf{Q} = (0.5{,}0.5{,}7)$ and (b) $\mathbf{Q} = (0.45{,}0{,}7)$ corresponding to the reduced phonon wave vectors $\mathbf{q} = (0.5{,}0.5{,}0)$ and $\mathbf{q} = (0.55{,}0{,}0)$, respectively [as shown in Fig. 1(a,b)], along with data taken at $T = 2\ \mathrm{K}$ (blue circles), i.e. well below the superconducting transition temperature $T_c = 15.2\ \mathrm{K}$. The blue lines are guides to the eye.

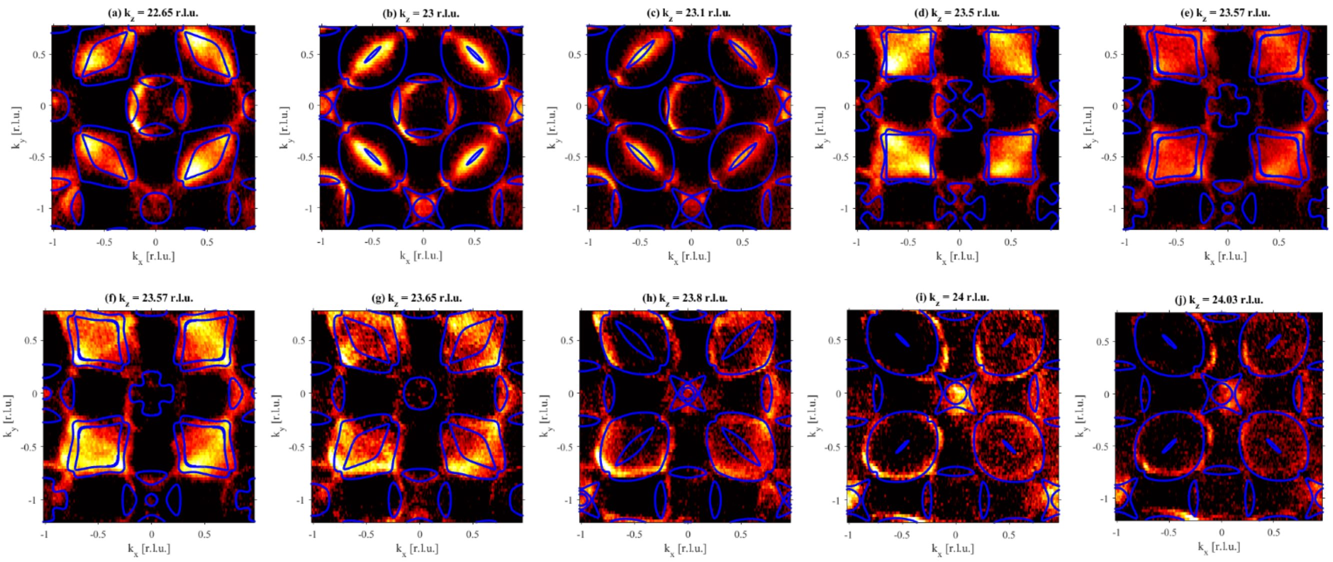

FIG. S2. Fermi surface cuts at constant k_z.

Comparison of calculated Fermi surface (lines) and SX-ARPES intensities observed in the $\Gamma - X/Z - M$ plane for the given values of k_z. The data from (a)-(j) were obtained with incident energies of (a) 670 eV, (b) 693 eV, (c) 700 eV, (d) 725 eV, (e) 730 eV, (f) 730 eV, (g) 735 eV, (h) 745 eV, (i) 758 eV and (j) 760 eV, respectively.

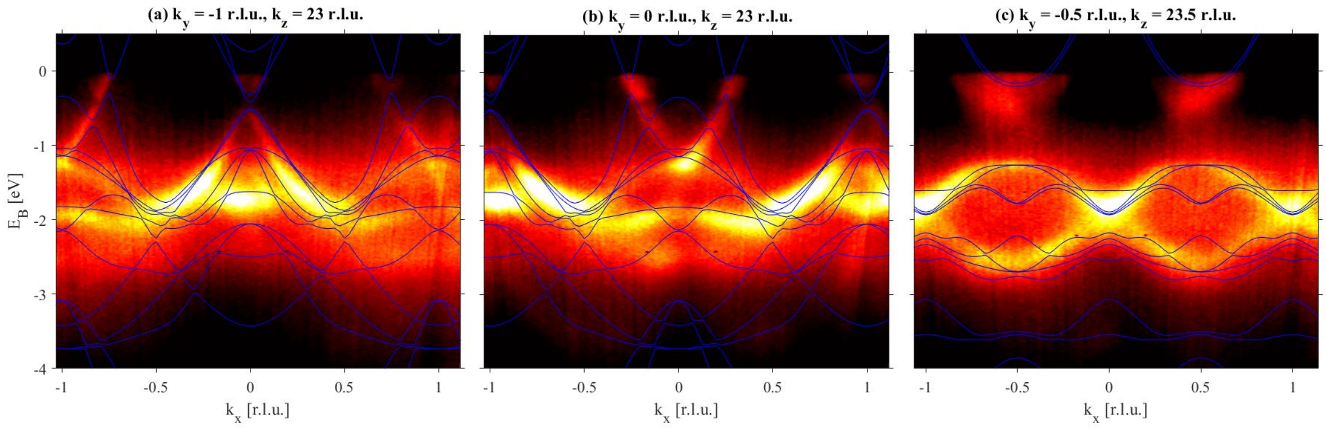

FIG. S3. Band structure for constant k_y and k_z.

Comparison of calculated band structure (lines) and SX-ARPES intensities for variable k_x at constant values of (a) $k_y = -1$ r.l.u. and $k_z = 23$ r.l.u., (b) $k_y = 0$ and $k_z = 23$ r.l.u., and (c) $k_y = -0.5$ r.l.u. and $k_z = 23.5$ r.l.u..

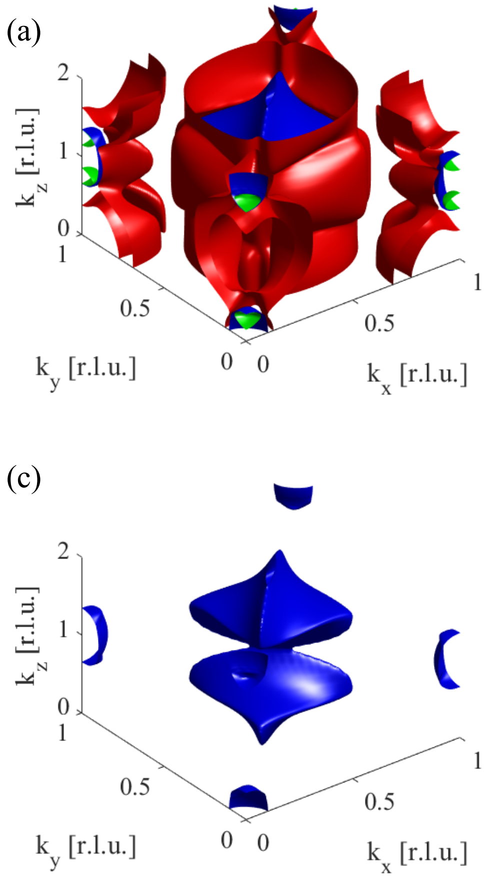

FIG. S4. Calculated Fermi surface.

(a) Complete calculated 3D Fermi surface. (b)-(d) Fermi surfaces for the three different electronic bands crossing the Fermi energy.

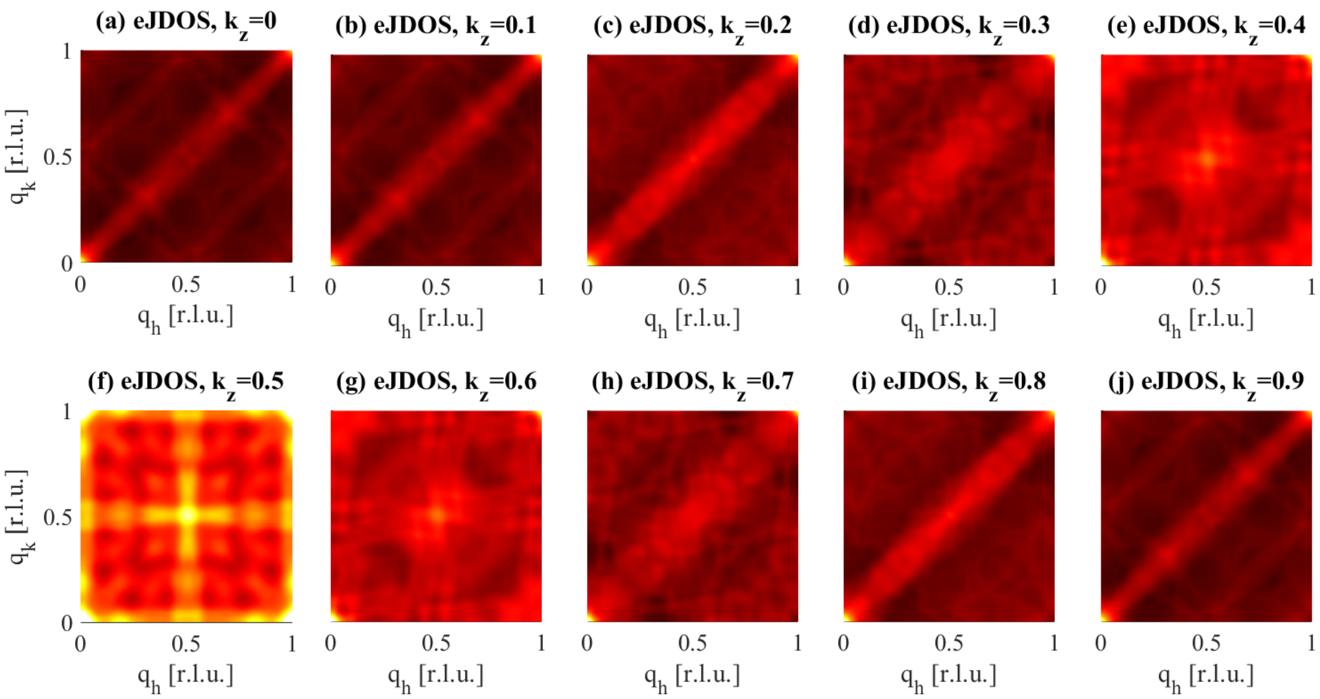

FIG. S5. Calculated 2D electronic joint density-of-states (2D-eJDOS) for fixed values of (a) $k_z = 0$, (b) 0.1 r. l. u., (c) 0.2 r. l. u., (d) 0.3 r. l. u., (e) 0.4 r. l. u., (f) 0.5 r. l. u., (g) 0.6 r. l. u., (h) 0.7 r. l. u., (i) 0.8 r. l. u., and (j) 0.9 r. l. u.. The color scale is the same as for Fig. 4(b)(c).

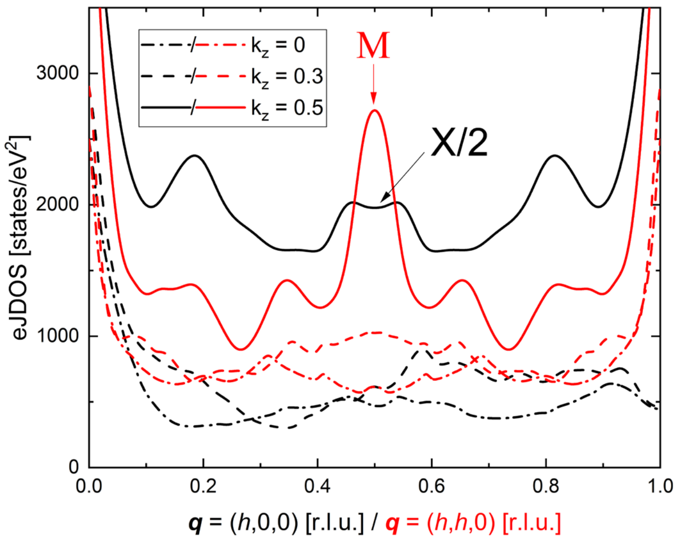

FIG. S6. Calculated electronic joint density-of-states (eJDOS) along two lines, i.e., $\mathbf{q} = (h, 0,0)$ (black lines) and $(h, h, 0)$ (red lines), $h = 0 - 1$, for fixed values of $k_z = 0, 0.3$ r. l. u. ,0.5 r. l. u. as dash-dotted, dashed and solid lines respectively.

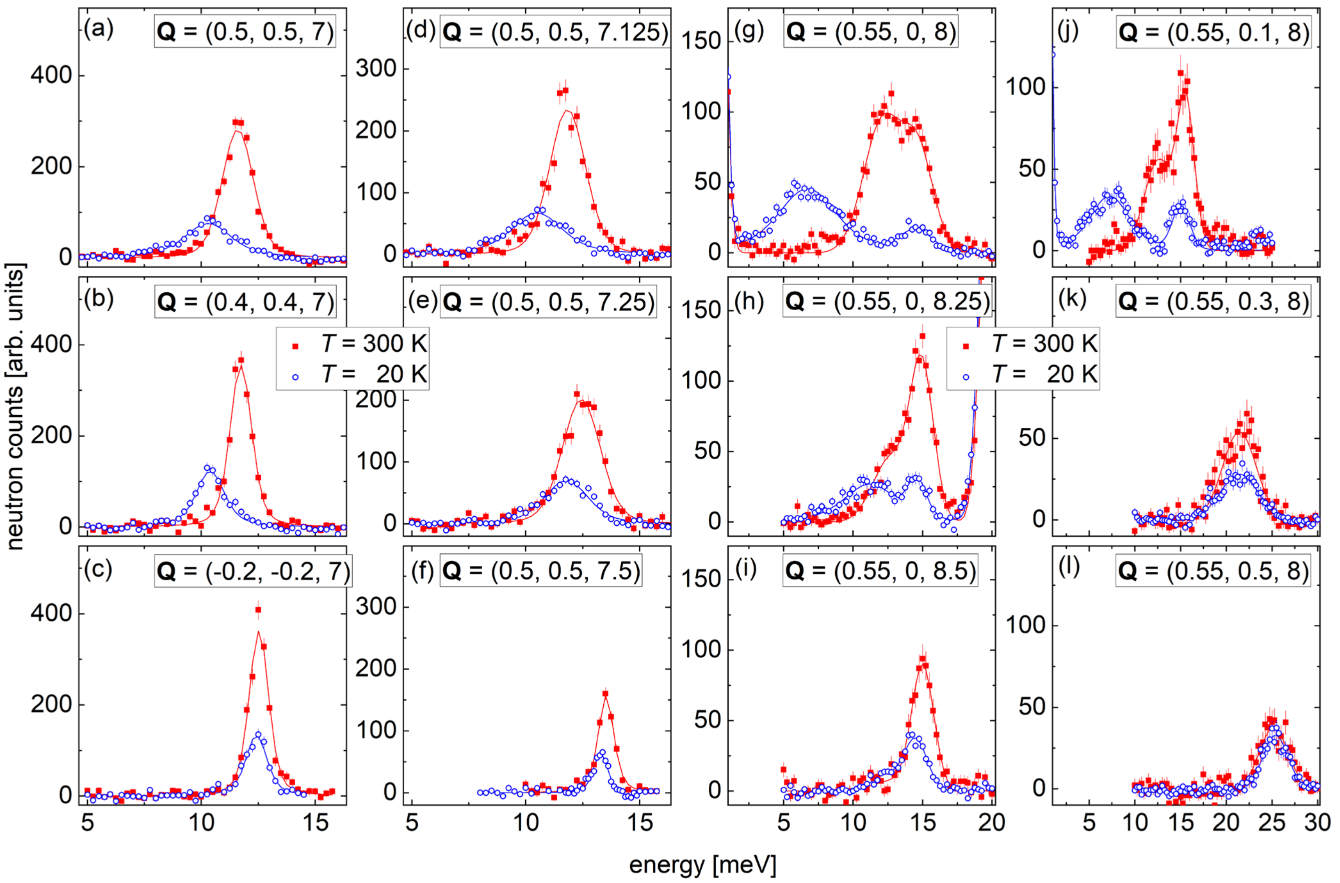

FIG. S7. Phonon spectroscopy at wave vectors away from high-symmetry directions.
Background subtracted scans in the vicinity of (a)-(f) $\mathbf{Q} = (0.5,0.5,7)$ and (g)-(l) $\mathbf{Q} = (0.55,0,8)$ at room temperature (red squares) and T = 20 K (blue circles). The respective wave vector values are given in the legend of each panel. Note that $\mathbf{Q} = (0.55,0,8)$ corresponds to the same reduced wave vector $\mathbf{q} = (0.55,0,0)$ [with $\boldsymbol{\tau} = (0,0,8)$] as $\mathbf{Q} = (0.45,0,7)$ [with $\boldsymbol{\tau} = (1,0,7)$]. Different to $\mathbf{Q} = (0.45,0,7)$, the first TO phonon has a finite structure factor at $\mathbf{Q} = (0.55,0,8)$ and is clearly visible in agreement with our structure factor calculations. Hence, the phonon softening shown in (g) is the same as that in Fig. 1(b) – but measured on the neutron spectrometer *1T* located at LLB, CEA Saclay, France.

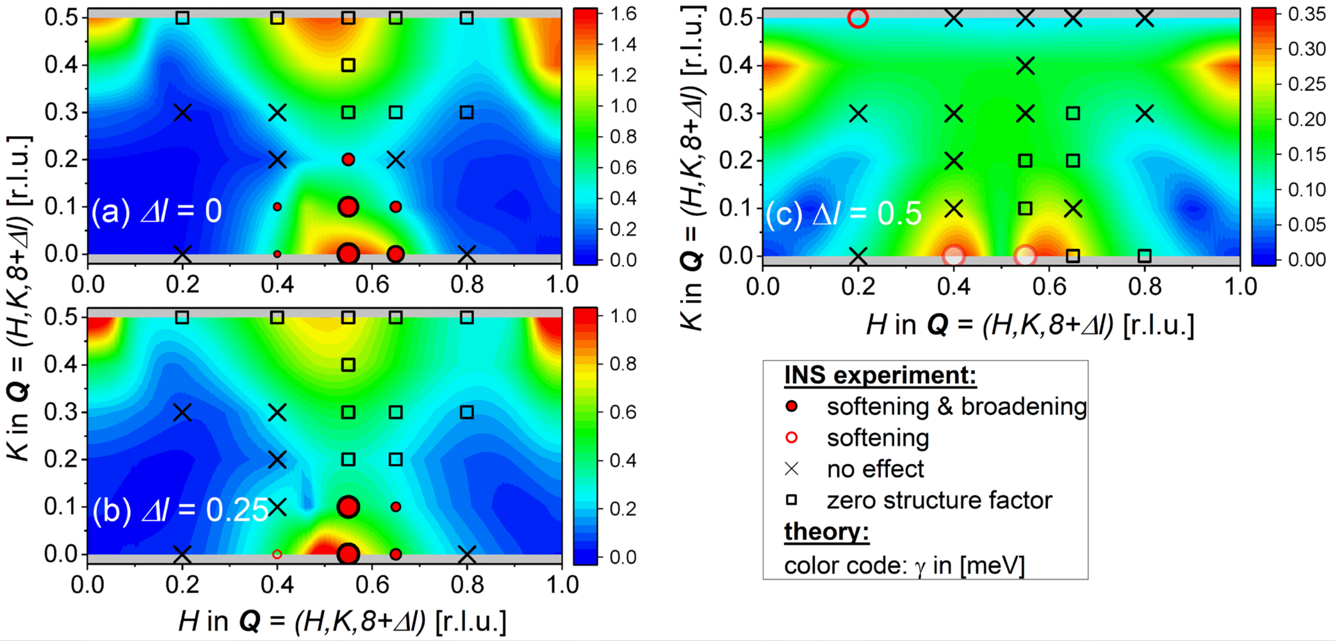

FIG. S8. Momentum dependent phonon renormalization near Q = (0.55,0,8).

Color-coded plot of the calculated electronic contribution to the phonon linewidth γ for the transverse acoustic phonon mode investigated close to $\mathbf{Q} = (0.55,0,8 + \Delta l)$. Results are shown in the (H,K) plane for (a) $\Delta l = 0$, (b) 0.25 r.l.u. and (c) 0.5 r.l.u.. Symbols refer to results from INS measurements and indicate phonon renormalization as indicated in the legend (lower right). Size of symbols (dots, circles) scale with the strength of the phonon renormalization within each panel.

Supplemental Note 1:

Opening of the superconducting gap 2Δ observed in phonon spectroscopy

Below we explain in some more detail the superconductivity-induced changes of the phonon line shape observed for the TA phonon modes in YNi_2B_2C both at $\mathbf{q} = (0.5,0.5,0)$ [Fig. S1(a)] and $\mathbf{q} = (0.55,0,0)$ [Fig. S1(b)]. Previously, some of us reported on this phenomenon in Ref. [1]. Similar studies for other superconductors can be found in Refs. [2-4].

Figure S1(a) shows the evolution through $T_c = 15.2$ K of the low temperature line shape of the TA phonon mode at the M point. On cooling from T = 300 K to 20 K, this phonon softens and broadens substantially, indicative of a strong EPC [see temperature dependences given in Figs. 1(c) and S1(c)]. However, the line shape remains Lorentzian to a very good approximation. On further cooling through T_c, the line shape starts to deviate strongly from a Lorentzian. In particular, a step-like increase at a certain energy $E_s \approx 5.7$ meV appears in the spectrum taken at T = 2 K [Fig. S1(a)]. According to the theory developed by Allen et al. [5], a part of the low energy tail which lies below the value of the superconducting gap 2Δ is pushed up in energy to form a narrow spike at 2Δ. The theory is based on the full quantum mechanical treatment of EPC where vibrational and electronic excitations mix into hybrid modes. The finite spectrometer resolution should wash out the theoretically predicted spike in experiments resulting in a sharp intensity increase at 2Δ. The predicted intensity increase corresponds to the one we observe at E_s, i.e. 2Δ(T=2K) = 5.7 meV for the TA phonon at ***q*** = (0.5,0.5,0). In Ref. [1], we were able to employ this signature of the superconducting gap in phonon spectroscopy in order to carefully determine the temperature dependence of 2Δ.

The phonon at ***q*** = (0.55,0,0) [Fig. S1(b)] has a significantly lower energy than the *M* point phonon discussed above [see Fig. 1(c)] and, moreover, has a much larger linewidth [Fig. S1(c)]. As a consequence, the superconductivity-induced redistribution of spectral weight is even stronger, but the superconducting gap cannot be inferred from the data as easily, except for temperatures close to T_c where the gap is still small. For lower temperatures, most of the spectral weight condenses into a fairly sharp peak [Fig. S1(b)] whose energy is – according to theory - somewhat below 2Δ. According to Ref. [5], this resonance can be regarded as a mixed vibrational or superelectronic collective excitation. Its position with respect to 2Δ has to be determined from calculations for the parameter values of this particular phonon. Again, the theory reproduces the observed line shapes sufficiently well to precisely determine the gap value.

For the current subject regarding the phonon renormalization in the normal state of YNi_2B_2C, the superconductivity-induced changes of the phonon line shape are important. They demonstrate that the linewidth at low temperatures of T ≈ 20 K is sensitive to changes of the electronic states at the FS and, thus, EPC in nature and not related to anharmonic broadening as observed in *2H*-$NbSe_2$ [6].

Supplemental Note 2:

Momentum dependent phonon renormalization

Phonon spectroscopy with INS is done at absolute wave vectors $\boldsymbol{Q} = \boldsymbol{\tau} + \boldsymbol{q}$, where $\boldsymbol{\tau}$ denotes the center of a Brillouin zone (BZ) and $\boldsymbol{q}$ is the reduced wave vector within this BZ. Phonon energies and linewidths are defined within the first Brillouin zone (BZ) and, thus, do not depend on $\boldsymbol{\tau}$. In contrast, the phonon intensity varies strongly with $\boldsymbol{\tau}$. For example, the TA mode at the *M* point discussed in our work has a large intensity at $\boldsymbol{Q}$ = (0.5,0.5,7) [Fig. 1(a)] but cannot be observed at (0.5,0.5,L) with L = 2,4,6.[7] We note that the accurate determination of phonon linewidths requires sizeable intensities. Thus, we could only evaluate the temperature dependence of the phonon energy in some cases with weak scattering intensities. In other cases, even this was not possible anymore.

Background subtracted INS data are shown in Figure S7. Measurements around the M-point were done at the IN8 triple-axis spectrometer, ILL, with Cu200 monochromator and Cu200 analyzer in order to achieve the best energy resolution. A fixed final energy of 14.7 meV was used and the sample was aligned in the horizontal 110-001 scattering plane. The data [Figs. S7(a)-(f)] visualize the phonon renormalization by cooling from room temperature (red dots) to 20 K (blue circles). Data at different positions along the [110] [Figs. S7(a)-(c)] and [001] directions [Figs. S7(d)-(f)] emphasize the evolution of the phonon softening and broadening which decrease moving away from the *M* point.

In addition to measurements within the horizontal 110-001 scattering plane, we performed scans also at out-of-plane wave vectors which were offset along the vertical $[1\bar{1}0]$ direction by $\Delta\boldsymbol{q}_1 = (0.1, -0.1, 0)$ and $\Delta\boldsymbol{q}_2 = (0.2, -0.2, 0)$. The scattering geometry is depicted in Fig. 5(a), where the horizontal 110-001 scattering plane as well as those offset by $\Delta\boldsymbol{q}_1$ and $\Delta\boldsymbol{q}_2$ are indicated by green (bottom), red (middle) and blue dashed lines (top). The corresponding results for the main scattering plane are shown in panel (b). Results in Fig. 5(b) are given in absolute wave vectors with the *M* point located in the lower right corner at $\boldsymbol{Q}$ = (0.5,0.5,7). We emphasize that the center of the BZ – corresponding to the M point at $\boldsymbol{Q}$ = (0.5,0.5,7) is $\boldsymbol{\tau}$ = (1,0,7) [see Γ point in Fig. 5(a)]. Hence, the line $\boldsymbol{Q}$ = (H,H,7) [horizontal line in bottom of Fig. 5(b)] corresponds to $\boldsymbol{q}$ =(0.5+h,0.5-h,0) with $h = 0 - 0.4$.

The detailed momentum dependence of the experimentally observed phonon renormalization is in good agreement with the predicted momentum dependence of the electronic contribution to the phonon linewidth γ, where the symbol size in Fig. 5(b) scales with the strength of the observed effects. Panels (c) and (d) show the analogue results but for wave vectors offset by (c) $\Delta\boldsymbol{q}_2 = (0.2, -0.2, 0)$ and (d) $\Delta\boldsymbol{q}_1 = (0.1, -0.1, 0)$. There is a clear shift of the phonon renormalization. In the main scattering plane [Fig. 5(b)] and the one offset by $\Delta\boldsymbol{q}_1$ [Fig. 5(d)] the effects are strongest at *H* = 0.5 [lower left corner in (b) and (d)], whereas the maximum effect for $\Delta\boldsymbol{q}_2$ occurs at *H* = 0.2 [Fig. 5(c)]. Here, we note that H = 0.2 corresponds in the notation of Fig. S8 to $\boldsymbol{Q}$ = (0.2,0.2,7) + (0.2,-0.2,0) = (0.4,0,7) and, thus, to $\boldsymbol{q} = \boldsymbol{Q} - \boldsymbol{\tau} = (0.4,0,7) - (1,0,7) = (-0.6,0,0)$. Hence, results with offset $\Delta\boldsymbol{q}_2$ are actually very close to the anomaly along the [100] direction on which we focused in our measurements taken close to $\boldsymbol{Q} = (0.55,0,8)$. However, we note that we used the very good energy resolution at the IN8 spectrometer to investigate the temperature dependence of the anomaly at $\boldsymbol{q} = (0.55,0,0)$ at an absolute wave vector of $\boldsymbol{Q} = (0.45,0,7)$. Corresponding results are shown in Fig. 2(b).

We investigated the momentum dependence of the phonon anomaly centered at $\boldsymbol{q} = (0.55,0,0)$ more extensively in the BZ adjacent to $\boldsymbol{\tau} = (0,0,8)$. We note that the bulk of the previous data for anomalous phonon scattering in YNi_2B_2C has been obtained at $\boldsymbol{Q} = (h, 0,8)$, $0 \leq h \leq 1$ [1,7-9].

Corresponding background subtracted INS data are shown in Figure S7(g)-(l). Measurements close to the anomaly at $\boldsymbol{Q}$ = (0.55,0,8) were done at the 1T triple-axis spectrometer, LLB, with PG002 monochromator and PG002 analyzer. A fixed final energy of 14.7 meV was used and the sample was aligned in the horizontal 100-001 scattering plane. The data [Figs. S7(g)-(l)] visualize the phonon renormalization by cooling from room temperature (red dots) to 20 K (blue circles). Data at different positions along the [001] [Figs. S7(g)-(i)] and the out-of-plane [010] directions [Figs. S7(j)-(l)] emphasize the evolution of the phonon softening and broadening which decrease moving away from $\boldsymbol{Q} = (0.55,0,8)$. The data also show the presence of higher energy phonon modes, e.g. an optic mode with an energy of 13-14 meV at $\mathbf{Q} = (0.55,0,8)$, the center of the anomaly in the TA mode [Fig. S7(g)]. This optic mode does not change with temperature as was already reported in previous work [7].

Results for the momentum dependent phonon renormalization observed in the BZ adjacent to $\boldsymbol{\tau} = (0,0,8)$ are summarized in Figure S8 for offsets in the l component of (a) 0, (b) 0.25 r.l.u. and (c) 0.5 r.l.u. from the zone center value. Again we find that the regions in reciprocal space with large calculated values of γ agree well with the points at which we observe detectable phonon renormalization. We emphasize that the fact that we did not observe phonon renormalization at some wave vectors for which large γ values are calculated, e.g. at the *M* point [$\boldsymbol{Q} = (0.5,0.5,8)$ in Fig. S8(a)], is solely due to a vanishing phonon structure factor of the TA mode at the *M* point in this BZ (marked by open square symbols) in agreement with calculations for the structure factor.

Supplementary references:

1 Weber, F., Kreyssig, A., Pintschovius, L., Heid, R., Reichardt, W., Reznik, D., Stockert, O. & Hradil, K. Direct Observation of the Superconducting Gap in Phonon Spectra. *Physical Review Letters* **101**, 237002 (2008).

2 Weber, F. & Pintschovius, L. Superconductivity-induced distortions of phonon lineshapes in niobium. *Physical Review B* **82**, 024509 (2010).

3 Weber, F., Rosenkranz, S., Heid, R. & Said, A. H. Superconducting energy gap of $2H-NbSe_2$ in phonon spectroscopy. *Physical Review B* **94**, 140504(R) (2016).

4 Sauer, A., Zocco, D. A., Said, A. H., Heid, R., Böhmer, A. & Weber, F. Electron-phonon coupling and superconductivity-induced distortion of the phonon lineshape in V_3Si. *Physical Review B* **99**, 134511 (2019).

5 Allen, P. B., Kostur, V. N., Takesue, N. & Shirane, G. Neutron-scattering profile of $\mathbf{Q} \neq 0$ phonons in BCS superconductors. *Physical Review B* **56**, 5552-5558 (1997).

6 Leroux, M., Errea, I., Le Tacon, M., Souliou, S.-M., Garbarino, G., Cario, L., Bosak, A., Mauri, F., Calandra, M. & Rodière, P. Strong anharmonicity induces quantum melting of charge density wave in *2H*-$NbSe_2$ under pressure. *Physical Review B* **92**, 140303 (2015).

7 Weber, F., Pintschovius, L., Reichardt, W., Heid, R., Bohnen, K. P., Kreyssig, A., Reznik, D. & Hradil, K. Phonons and electron-phonon coupling in YNi_2B_2C. *Physical Review B* **89**, 104503 (2014).

8 Kawano, H., Yoshizawa, H., Takeya, H. & Kadowaki, K. Anomalous phonon scattering below T_c in $YNi_2{}^{11}B_2C$. *Physical Review Letters* **77**, 4628-4631 (1996).

9 Zarestky, J., Stassis, C., Goldman, A., Canfield, P. C., Shirane, C. & Shapiro, S. Phonon profiles in superconducting YNi_2B_2C and $LuNi_2B_2C$. *Physical Review B* **60**, 11932-11934 (1999).
